# Can bovine TB be eradicated from the Republic of Ireland? Could this be achieved by 2030?

**DOI:** 10.1186/s13620-019-0140-x

**Published:** 2019-04-25

**Authors:** Simon J. More

**Affiliations:** Centre for Veterinary Epidemiology and Risk Analysis, UCD School of Veterinary Medicine, University College Dublin, Belfield, Dublin, D04 W6F6 Ireland

**Keywords:** Bovine tuberculosis, Ireland, Eradication, Constraints, Wildlife risks, Risk-based cattle controls, Industry engagement

## Abstract

**Background:**

There has been an ongoing decline in bovine tuberculosis (TB) in the Republic of Ireland, however, TB has yet to be eradicated. Further to a recent commitment by the Irish government to eradicate TB by 2030, this paper considers two questions, ‘Can bovine TB be eradicated from the Republic of Ireland?’ and ‘Could this be achieved by 2030?’, given current knowledge from research.

**Main body of the abstract:**

Until very recently, Ireland has lacked key tools required for eradication. This gap has substantially been filled with the national roll-out of badger vaccination. Nonetheless, there is robust evidence, drawn from general national research, international experiences, and results of a recent modelling study, to suggest that all current strategies plus badger vaccination will not be sufficient to successfully eradicate TB from Ireland by 2030. We face a critical decision point in the programme, specifically the scope and intensity of control measures from this point forward. Adequate information is available, both from research and international experience, to indicate that these additional measures should broadly focus on adequately addressing TB risks from wildlife, implementing additional risk-based cattle controls, and enhancing industry engagement. These three areas are considered in some detail.

**Conclusion:**

Based on current knowledge, it will not be possible to eradicate TB by 2030 with current control strategies plus national badger vaccination. Additional measures will be needed if Ireland is to eradicate TB within a reasonable time frame. Decisions made now will have long-term implications both in terms of time-to-eradication and cumulative programme costs.

## Introduction

**Fig. 1:**
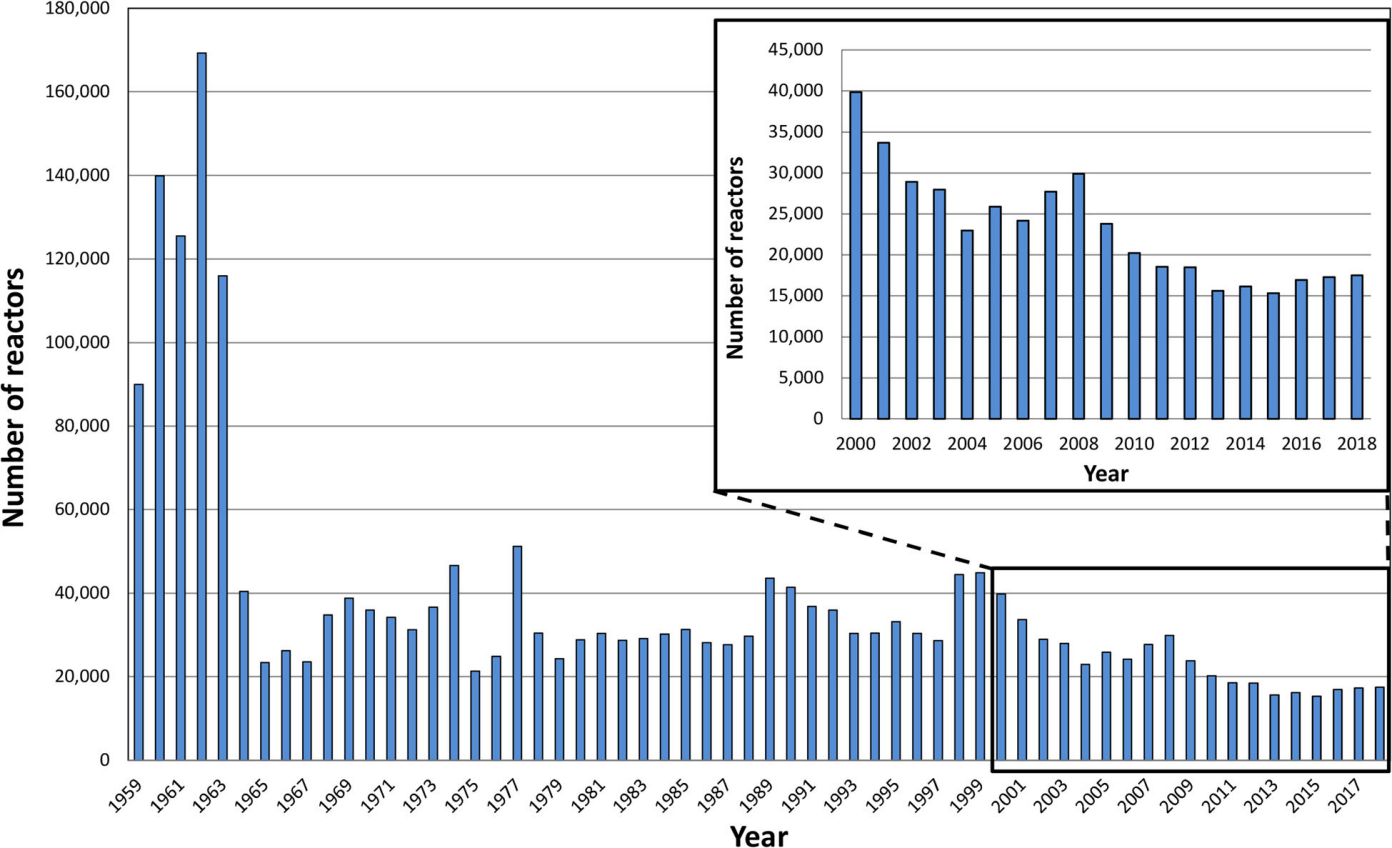
The annual number of TB reactors in Ireland, from 1959 (when records are first available) to 2018, including magnification of the period from 2000 to 2018

There has been an ongoing decline in bovine tuberculosis (TB, caused by infection with *Mycobacterium bovis*) in the Republic of Ireland (subsequently termed Ireland), although reactor numbers have remained steady in recent years (Fig 1). Comparisons of TB incidence in Ireland and the countries of the UK has been published [1, 2]. Although good progress is being made, TB has yet to be eradicated from Ireland.

The national TB eradication programme is informed by detailed ongoing research, conducted by a number of different research groups including the Centre for Veterinary Epidemiology and Risk Analysis (CVERA) at University College Dublin. Since its establishment in 1989, the TB-related research conducted by CVERA (previously the Tuberculosis Investigation Unit) has focused on two broad issues, including an improved understanding of constraints to national eradication, and practical solutions to address these constraints. In broad terms, research has addressed three key areas including cattle (with the objectives of improving detection of infected herds, improving clearance of TB from infected herds), wildlife (clarifying the role played by badgers in TB infection in cattle, gaining an improved understanding of badger ecology and TB epidemiology in this species, identifying appropriate control strategies to limit infection in badgers and to cattle), and the overall programme (evaluating appropriate models of governance and cost-sharing, gleaning lessons from international experiences of success and failure). The national programme has evolved substantially over time in response to new knowledge.

On 8 May 2018, the Irish government approved a proposal from the Minister for Agriculture, Food and the Marine, Michael Creed TD, to commit to the eradication of TB by 2030. This commitment has been supported by the establishment of a Bovine TB Stakeholder Forum tasked with proposing policies to help achieve eradication within this timeframe [3]. The national target does not distinguish between biological freedom (extinction of *M. bovis* from Ireland) and legal freedom (which includes regular testing of all herds with no evidence of infection during the previous three years in at least 99.8% of herds representing at least 99.9% of bovids in the country or zone) [4].

This paper considers two questions, ‘Can bovine TB be eradicated from the Republic of Ireland?’ and ‘Could this be achieved by 2030?’, given this context and based on current knowledge from research.

## Are we doing enough to successfully eradicate TB from Ireland by 2030?

Until very recently, Ireland has lacked key tools required for eradication, including the ability to sustainably prevent the spread of infection from wildlife to cattle. In such circumstances, it has been appropriate to control TB as effectively as possible (essentially a progressively improving ‘holding pattern’) whilst seeking to fill critical gaps in knowledge. This gap has substantially been filled as a consequence of research on the utility of badger vaccination to limit the transmission of infection within badger populations and the spread of infection from badgers to cattle [5–15]. Therefore, the ongoing roll-out of badger vaccination is a very important addition to the national programme.

Even with this addition, however, there is robust evidence to suggest that all current strategies plus badger vaccination will not be sufficient to successfully eradicate TB from Ireland by 2030. This evidence is drawn from general national research, international experiences, and results of a recent modelling study.

### General national research

As part of the large body of research conducted in Ireland, a number of challenges have been identified, including some that may substantially constrain progress towards eradication. These include:

- *Aspects of the disease itself*, including the presence of residual infection (infected animals that test negative to current diagnostic tests) and the prolonged (but variable) period of heightened risk that occurs in herds following infection,
- *The presence of a multi-host system (that is, cattle and badgers),* which requires a multi-faceted strategy to adequately control infection in all animal species of epidemiological relevance (that is, animal species that contribute to both the maintenance and spread of TB infection in Ireland),
- *Programme fatigue,* noting that there have been ongoing eradication effort since the late 1950s,
- *Commercial realities*, including both the substantial and ongoing movement of cattle in Ireland and the need for minimal disruption from the programme to allow ongoing commerce, and
- *Limited industry engagement*, as reflected in the current models of programme governance and cost-sharing.

### International experience

TB has been successfully eradicated from only a small number of countries, primarily Australia and several countries in northern Europe. There has been close collaboration between scientists and policy-makers in a range of affected countries, with the international *M. bovis* conferences (1st in Dublin in 1991^1^; 2nd in Dunedin in 1995; 3rd in Cambridge in 2000^2^; 4th in Dublin in 2005^3^; 5th in Wellington in 2009^4^; 6th in Cardiff, Wales in 2014; 7th to be held in Galway in 2020) being one opportunity to share experiences. There are lessons to be learned from other countries that are of potential benefit to Ireland. With respect to countries facing similar experiences, efforts towards eradication have been lengthy in Ireland, but also in Australia (a 27-year programme [16, 17]) and in New Zealand and the UK (many decades). Similarly, wildlife contribute (or have contributed) to the epidemiology of TB in many countries, including Ireland, but also Australia (feral buffalo and feral pigs) [16], France (badgers, deer, wild boar) [18], New Zealand (brush-tailed possum) [19], Spain (wild boar and deer) [20], UK (badgers) [21] and USA (white-tailed deer in Michigan) [22].

Lessons learned from the successful eradication of TB from Australia have been documented [16]. In comparison with Australia (where eradication has been successful) or New Zealand (where substantial progress is being made), there are clear differences, as outline below, in the Irish programme in terms of cattle controls and industry engagement.

### Results from a recent modelling study

In partnership with Wageningen University (the Netherlands), work has recently been completed within CVERA to assess the effectiveness of current control strategies to achieve biological eradication of TB from cattle and badgers in Ireland, both prior to and following the inclusion of badger vaccination [9]. Central to this work is the concept of the ‘reproduction ratio’ (termed *R*), this being the average number of secondary cases caused by each primary case. An epidemic can only be sustained if *R* is greater than one. Therefore, the efficacy of control measures can be assessed based on whether or not they are capable of reducing *R* below one. Therefore, *R* = 1 could be considered equivalent to the *‘threshold for biological eradication of M. bovis from Ireland’.* For values of *R* below 1, time-to-eradication will shorten as *R* decreases (that is, as *R* is reduced well below 1).

The key results from the Wageningen-CVERA study suggest that eradication would not have been achieved with all current control strategies (that is, prior to the introduction of badger vaccination). In these circumstances, it is estimated that *R* for the cattle-badger system lies between 1.07 and 1.16, depending on the assumptions used. Following the introduction of badger vaccination in addition to all current control strategies, *R* for the cattle-badger system will be reduced below 1, but not substantially (that is, *R* = 0.93–0.97). These latter estimates assume national badger vaccination coverage of 40% and national average badger TB prevalence of 14%. *R* for the cattle-badger system would decrease with higher levels of national badger vaccination coverage and increase with higher national average badger TB prevalence. These estimates also come with a number of points of caution, noting that each has the potential to increase *R* for the overall system, with implications for the feasibility of biological eradication given badger vaccination in addition to current control strategies. Specifically, the modelling work currently only considers a two-host system (cattle, badgers). The work is based on national averages (eg cattle herd and badger TB prevalence), therefore *R* would be expected to vary in different areas even though levels of badger vaccination coverage might be equivalent. In addition, badger densities will rise with the ongoing shift from culling to vaccination, which would likely lead to a rise in *R*. Finally, these calculations assume a vaccine efficacy for susceptibility (VE_s_) of 0.59, but do not consider the associated uncertainty. The current estimate of VE_s_ from the Kilkenny badger vaccination study is 0.59 (95% confidence interval: 0.065–0.82) [10].

From this work, we conclude that TB eradication may be achievable with the addition of badger vaccination to all current control measures, however, it will take a very long time (that is, many decades). Further measures will be needed, in addition to current controls plus badger vaccination, if Ireland is to eradicate TB within a reasonable time frame.

## A critical decision point

We face a critical decision point in the programme, specifically the scope and intensity of control measures from this point forward. Decisions made now will have long-term implications in terms of both time-to-eradication (including whether the 2030 target is at all realistic), and the cumulative cost of the eradication programme, from now to the point of eradication and beyond.

**Fig. 2:**
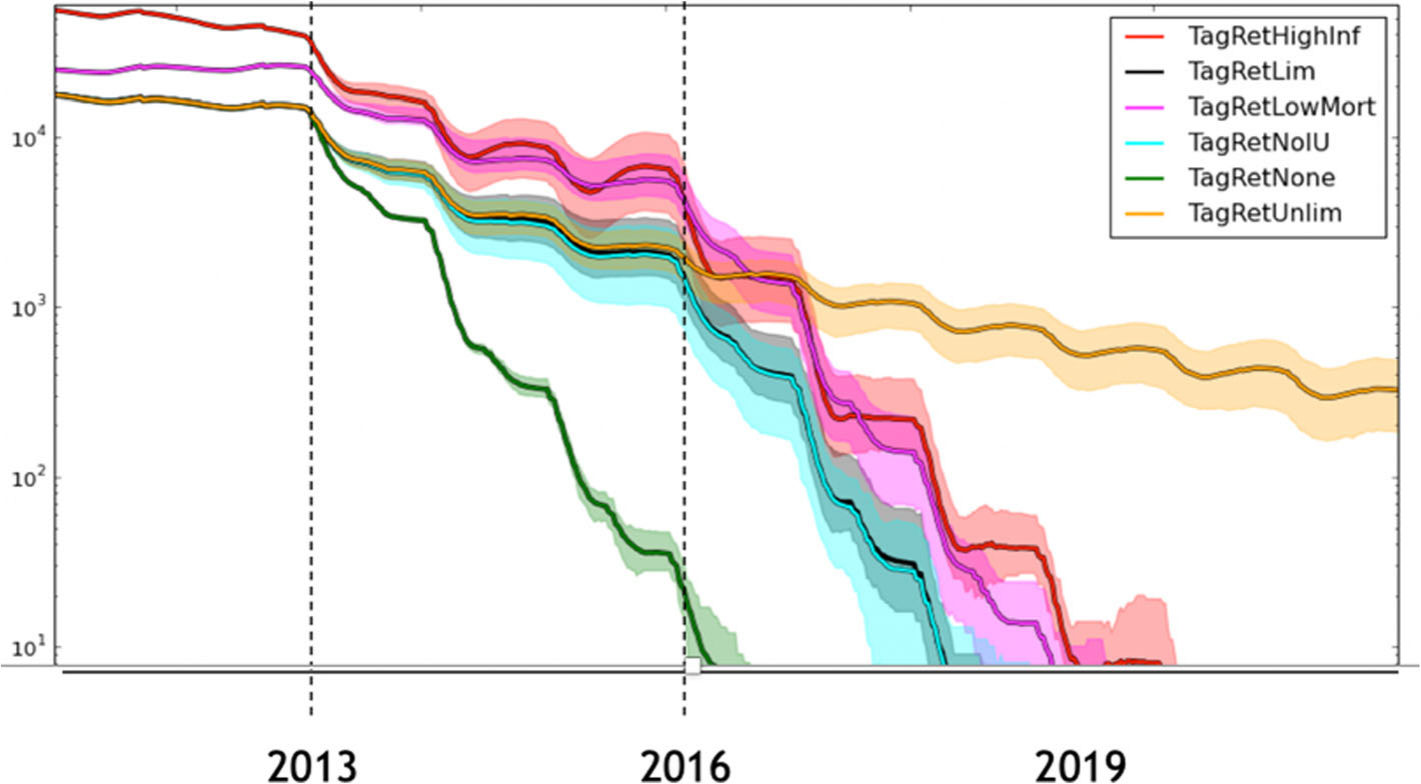
Estimated total number of herds infected with bovine viral diarrhoea (BVD) virus (y-axis, log scale) in Ireland in the years following the start of the compulsory national BVD eradication programme, under differing levels of retention of persistently infected (PI) animals. Output of the Irish BVD model (FarmNet 1.0) as of 2015. For modelling details see Thulke et al. [22] or http://www.ecoepi.eu/FarmNet-BVD/. In particular note: *The green line (TagRetNone)*: the predicted fall in total PI numbers assuming all PIs are removed from farms immediately following testing (that is, without any PI retention). Under this scenario, estimated time to eradication is 3–4 years from programme start (2016–17). *The yellow line (TagRetUnlim):* predicted fall in PI numbers given high levels of PI retention (that is, PI retention continues at a high level each year). Under this scenario, eradication is unlikely to occur. *The black line (TagRetLim,* generally obscured by the light blue line*):* the predicted fall in PI numbers, assuming high levels of PI retention during the first three years of the programme, but no PI retention subsequently. Under this scenario, estimated time to eradication is 6–7 years from programme start (2019–20). Three additional lines were included to test sensitivity assumptions, including: *the red line* (*TagRetHighInf,* doubling of transmission probabilities), *the purple line* (*TagRetLowMort,* doubling of survival time of PI animals) and *the light blue line* (*TagRetNoIU,* suppression of movement of animals with in utero infections)

This decision point is well illustrated from experiences gained in the national bovine viral diarrhoea (BVD) eradication programme, which is another animal disease eradication programme in Ireland, coordinated by Animal Health Ireland. The long-term impact, in terms of time-to-eradication, of the retention of BVD persistently infected (PI) animals on Irish farms is presented in Fig. 2 [23].

## Additional measures

The aforementioned Wageningen-CVERA modelling study [10] has highlighted the need for further measures, in addition to all current controls and badger vaccination, if Ireland is to eradicate TB within a reasonable time frame. Drawing on research findings, international experience and a detailed understanding of the situation in Ireland, it is my view that this is best achieved by adequately addressing TB risks from wildlife, implementing additional risk-based cattle controls, and enhancing industry engagement.

### Adequately addressing TB risks from wildlife

#### Badgers

Based on available evidence (including [7, 9, 10]), a national programme of badger vaccination will substantially contribute to national eradication efforts. There is a need for ongoing critical evaluation of this programme, investigating both the dynamics of TB infection in badgers and changes in the risk posed to cattle. Particular emphasis should be placed on the non-inferiority trial (where comparison is being made between badger vaccination and ongoing badger culling), detailed monitoring and evaluation of ongoing badger vaccination particularly in areas where problems arise, and relevant aspects of badger ecology. Each of these issues is an area of active national research.

#### Wild deer

When considering the role of wild deer, and indeed of other wildlife species, it is important to note the differing ‘epidemiological roles’ that infected wildlife can play with respect to TB in cattle. Specifically, wildlife species can act either as a spillover host, a maintenance host or a maintenance host with spillback to cattle, noting that a maintenance host is defined as a wildlife species in which infection is self-sustained in that species [24, 25]. A spillover host is likely of little concern for national TB eradication, whereas wildlife that act as a maintenance host with spillback to cattle, such as badgers in Ireland, pose substantial challenges. As one example, during the Australian TB eradication programme, feral pigs (an invasive species in that country) became infected whilst scavenging on infected cattle carcasses. However, infection was not maintained in these populations, and it disappeared from feral pigs once it had been eliminated from cattle [24]. This information is important, as TB eradication would have proved very difficult if feral pigs had been a maintenance host. Currently, there are an estimated 24 million feral pigs in Australia, roughly equivalent to the human population [26].

In some countries, there is evidence that wild deer act as a maintenance host, playing an important role in the epidemiology of TB in cattle. In Spain, in some populations of red deer (*Cervus elaphus*), TB has been found at high prevalence (up to 50%), with more than 50% of infected animals presenting with generalised infection [27]. Based on detailed work conducted over many years, in one region of Michigan (USA) white-tailed deer (*Odocoileus virginianus*) are recognized as a maintenance host for TB, posing an ongoing TB risk to neighbouring cattle [28–31]. Several factors were crucial to the establishment of self-sustaining TB in this deer population, including intensive baiting and supplementary feeding of deer during winter [28]. It is well recognised that increased population density and population aggregation each facilitate TB transmission. In New Zealand, transmission within wild deer populations is rare, and wild deer are not recognised as maintenance hosts for TB. However, transmission from wild deer carcasses to scavengers, including brush-tailed possums (*Trichosurus vulpecula*), can occur, creating a ‘spillback risk’ that could persist for some years after transmission of new infection to wild deer has been halted [32].

In Ireland, data are sparse, and the epidemiological role played by wild deer (predominantly Sika (*Cervus nippon*) or Sika hybrids) is currently uncertain:

- Using occurrence data (that is, presence or absence in defined areas, based on confirmed deer sightings), Carden et al. [33] found a considerable expansion in the range of several deer species in Ireland between 1978 and 2008. Trends in deer density are not available.
- Based on available data (all unpublished, except [34, 35]), TB prevalence in wild deer is very low in most areas of Ireland. Based on the results of passive surveillance of deer – that is, wild deer which were shot and submitted to Regional Veterinary Laboratories for TB testing – from areas outside County Wicklow during 2017 and 2018, 73 wild deer were tested and three were reported to have had TB (4.1%) (unpublished). Of 17 wild deer that were examined during a large outbreak of TB in north Co. Sligo, none were found to be infected [35].
- Higher TB prevalence has been observed in several hot-spot areas of Co. Wicklow (those with high TB prevalence in cattle). An unpublished research study carried out by the national Department of Agriculture, Food and the Marine (DAFM) in the Calary area of Wicklow in 2014 and 2015 found that 16% of deer had TB in that area. Sampling was non-random, using fresh full carcasses. The same (local) TB strain was identified in cattle, badgers and deer. An ongoing follow-up study by DAFM, from the same area, recently reported that 8.3% (10/121) of deer shot on farmland had TB, whereas 0% (0/32) of deer from a nearby control area (in the local National Park) were infected. Sampling was again non-random, but using frozen heads and plucks.

In most areas of Ireland, there is no evidence in support of deer acting as a maintenance host for TB. In hot-spot areas of Co. Wicklow, the epidemiological role played by deer is uncertain. Higher TB prevalence has been observed, however, this does not provide conclusive evidence that TB is self-sustaining in local deer populations, nor – if it is – of the relative contribution of infected deer to local TB epidemiology (establishment and spread).

Clarifying the epidemiological role of wildlife species is not straightforward [24, 25, 36], and the methodologies used in Michigan are not directly transferable to Ireland. Two recommendations are made, relevant to Ireland. In geographical areas of concern, deer should be managed to minimise risk factors that are known to facilitate the establishment and perpetuation of deer as a maintenance host for TB. Based on international experience and general principles, these risk factors include increased population density and circumstances that facilitate aggregation (both of deer *per se*, and of deer with other known infected species). Concurrently, deer removed during these management operations should be utilized to maximise their scientific value in clarifying the epidemiological role being played by deer in these localities. Using this material, and building on earlier research, it is appropriate to conduct ecological and epidemiological studies to address questions relevant to TB establishment, pathogen transmission/spread/persistence, both within and between relevant species (cattle, badgers, deer), and laboratory studies (pathology, microbiology) to further clarify the natural history of infection in this species (including route(s) of infection, the anatomical location of lesions, the route(s) and levels of excretion) [24]. Emerging technologies, including whole genome sequencing (WGS), may assist in tracking the TB-causing pathogen in time and space, to determine the direction and relative frequency of spread between cattle, badgers and deer in the same locality [37, 38]. WGS has been used in a number of settings relating to TB in cattle and wildlife, including Germany (in a wildlife park [39]), New Zealand [40, 41], UK [42] and the USA [43] (in the latter three countries, as part of their national TB eradication programme). WGS is currently being applied to TB samples from cattle, badgers and deer in the Calary area of Co. Wicklow, seeking a better understanding of the epidemiological role played by deer in this locality.

There is currently no evidence that TB is maintained in other farmed and wild animal species in Ireland, such as goats.

### Implementing additional risk-based cattle controls

#### TB herd risk

In endemic countries (where TB is present), it is not possible using current technologies to determine with 100% confidence whether a herd is TB infected or not. Rather, it is more appropriate to consider herds to be at differing levels of TB risk, from very low to very high. Infected herds are at greater TB risk for an extended period (up to 10 years) after TB derestriction (that is, following release after a TB restriction) [44], depending on factors including the size of the initial breakdown, herd size and herd location [45, 46]. Persistent TB risk contributes to herd recurrence and local persistence of TB [45].

There are two main drivers of persistent TB herd risk including infection in the locality (associated with neighbouring cattle and local wildlife) and infection in the herd (due to residual infection) [45].

#### Persistent TB herd risk due to residual infection

Residual infection refers to the presence of infected – but undetected – animals. Most of these animals are undetectable using available tests, either due to latent infection or anergy [47, 48]. This is of particularly concern at the time of TB derestriction, noting that residually infected animals can pose a future infection risk to the index or neighbouring herds, or to herds to which the animal subsequently moves. Multiple studies from a range of countries have highlighted the contribution of residual infection to TB persistence in a herd or locality (including [16, 45, 49, 50]). Further, difficulties in clearing infected herds, leading to herd TB recurrence, has been identified as a key challenge to TB eradication, in Ireland [45], New Zealand [50] and the UK [51].

The problem is essentially technical but exacerbated by current legislation. It is not possible with current diagnostic tools (including the use of interferon-*γ*) to identify all infected animals within known infected herds. Further, under relevant EU legislation [52], restricted herds are free to trade (and considered at no greater risk than non-infected herds) once two consecutive clear full-herd skin tests are achieved. In other words, herds are free to trade within 4 months after the last known infected animal has been detected. For comparison, in the successful Australian programme, all animals present during a breakdown were considered at risk for the rest of their life, and infected herds took a minimum of 8 years to attain the lowest herd risk status. Towards the latter stages of the programme, when infected herds were identified, there was a shift from ‘test and slaughter’ to whole herd depopulation to eliminate the threat posed by residual infection [16]. The EU legislation does not adequately mitigate the aforementioned heightened TB risk associated with these herds.

#### Cattle movement leading to ongoing recycling of infection

**Fig. 3:**
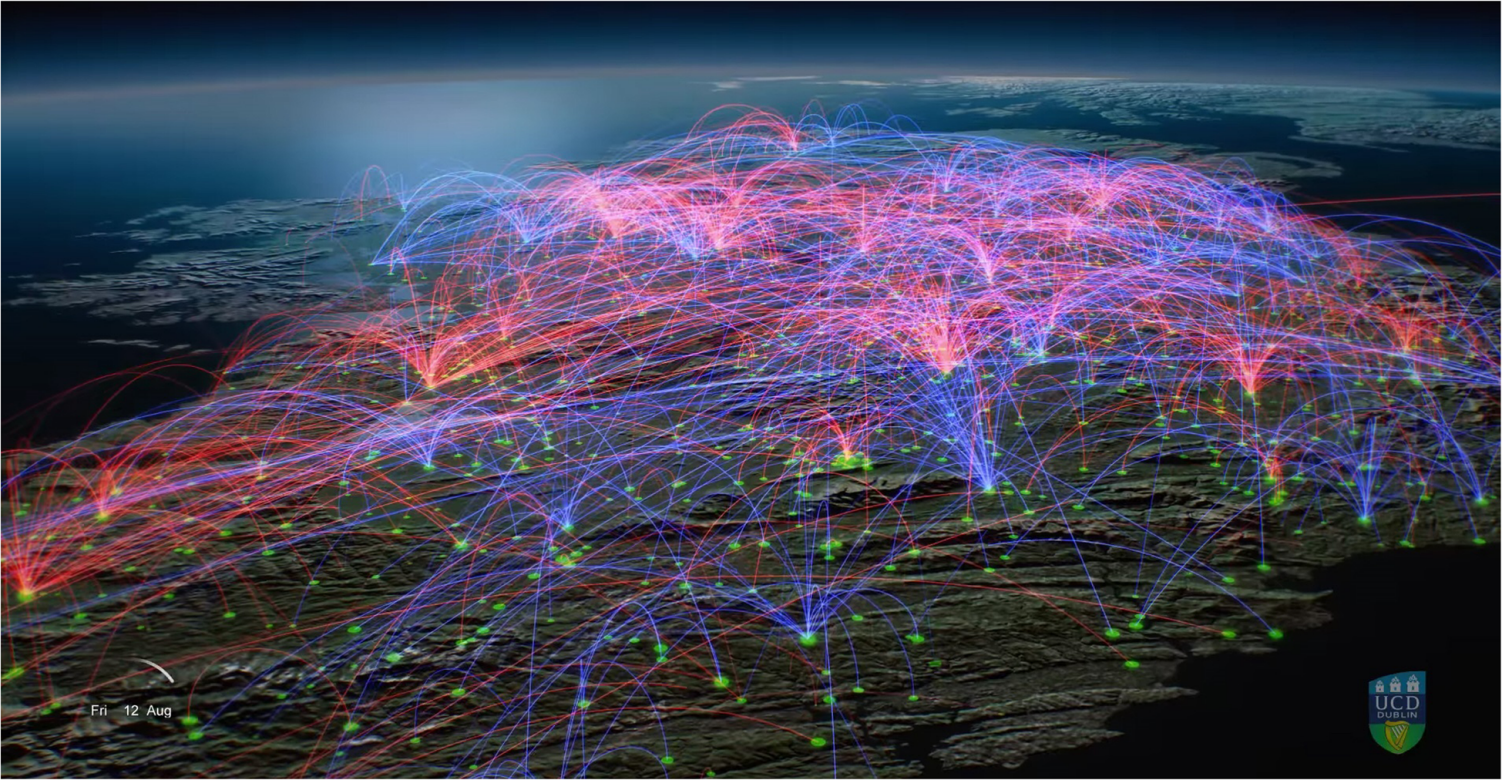
Screenshot of cattle movement events in Ireland, in this case from 12 August 2016. From McGrath et al. [50]. The blue lines depict movements to slaughter or export, and the red lines from farm to farm including via a mart. The movement video is available on YouTube at https://youtu.be/PTCdPMnenBw

There is very substantial movement of cattle in Ireland. In 2016, there were 1.3 million movement events, this being all journeys travelled by vehicles (such as trailers) to transport cattle to marts, new herds, slaughter plants or export facilities. These movement events covered a cumulative distance of 46 million kilometres in a single year (equivalent of circumnavigating the Earth 1015 times or travelling to the moon and back 60 times) [53] (Fig. 3).

The problem of residual infection coupled with substantial cattle movement leads to ongoing ‘churn’ or recycling of infection within the national population. This problem will greatly constrain efforts towards successful TB eradication. Further, the relative importance of this problem will increase as other infection sources are addressed.

This conclusion is not at odds with earlier Irish work, based on data from 2003 to 04 [54] and 2012 [44], attributing 6–8% of TB restrictions to the recent introduction of an infected animal. In endemic situations, as in Ireland, it has proved very difficult to disentangle the relative contribution of different infection sources using current epidemiological methods [45]. In Ireland, this was first achieved by White et al. [55] who focused on the relative importance of ‘neighbourhood’ in TB persistence, specifically farm-to-farm spread and spread from wildlife. In the two above-mentioned studies where TB restrictions were associated with the recent introduction of an infected animal [44, 54], source attribution was determined after considering the past movement history (including potential for TB exposure) of animals identified as reactors at the start of a TB restriction. However, there are several reasons why these estimates must be interpreted with caution. On the one hand, ‘potential for exposure’ was assumed to lead to infection, if this is not always the case risk has been overestimated; conversely, potential for latency (animals becoming infected following exposure but passing at least one test following introduction) was not considered, if it were important, risk has been underestimated [54]. Three different approaches have been used to overcome these concerns, although none has yet in Ireland. Firstly, modelling studies have been used in the UK to quantify source attribution in TB restrictions. In one study, 16% of TB restrictions were attributed to cattle movement [56] whereas another study suggested 13% attributed to cattle movement alone plus 40% to the combined effect of movement, transmission from the environment (including wildlife), and residual infection [57]. Secondly, WGS has been used in several countries to assist with source attribution [40–43]. Finally, in Australia, source attribution (both cattle movement and residual infection) become increasingly clear during the latter stages of the eradication programme as case numbers fell [16].

#### A risk-based approach

A risk-based approach is currently the only method available internationally to adequately address the problems caused by residual infection and animal movement, whilst also facilitating ongoing commerce within the farming community. This approach was central to the national eradication programmes in Australia [16] and New Zealand [58] and was also recently recommended for introduction in Great Britain [59]. Using this approach, TB risk is assessed at the level of the herd (not the animal), with herds progressively moving from a high TB herd risk score (at the time of derestriction) to a low TB herd risk score over a series of years. A broad range of measures are used to assist high TB risk herds to clear infection, and risk-based trading allows ongoing commerce whilst limiting the potential for infection to spread from herds of higher to lower TB risk through animal movement. This is achieved by allowing farmers to sell cattle to herds of equivalent or higher TB herd risk and to source cattle from herds of equivalent or lower TB herd risk [16].

### Enhancing industry engagement

In Ireland, TB is widely considered a government problem. This is in contrast to international examples of success, where TB eradication has been very reliant on models of programme governance/management and cost-sharing that encourage high level of industry engagement. In the successful Australian TB eradication programme, one commentator suggested that the programme ‘enjoy[ed] industry “ownership” and involvement at all levels of management’ [16]. Another indicated that ‘the involvement of industry in both funding and policy development was an essential factor in achieving the outcome of the campaign’ [17]. The Australian TB eradication programme led to the formation of Animal Health Australia (https://www.animalhealthaustralia.com.au) which now coordinates and facilitates many aspects of national animal health in Australia. In New Zealand, governance of the national TB eradication programme is overseen by OSPRI (https://www.ospri.co.nz), a non-government organization that manages both TB free New Zealand and NAIT (the national animal identification and traceability system).

Cost-sharing by government and industry has been a key feature of both the Australian and New Zealand programmes, although different models are used. In Australia, the programme was funded 50:50 by government (Federal and State) and industry, with the latter funded through a cattle transaction levy [60]. High level decision-making reflected the cost sharing formula, with government (both Federal and State) and industry involved. If TB were ever to recur (the last known TB case in Australia was in 2002), a cost-sharing model of 20:80 (government:industry) has been legally agreed [61], reflecting a shared understanding of the perceived public:private good associated with this disease. In New Zealand, cost-sharing is guided by principles outlined in national biosecurity legislation (Biosecurity Act 1993), with cost-sharing allocated after identifying both the beneficiaries (who will benefit from the control/eradication efforts) and the exacerbators (who is perpetuating the problem, essentially constraining eradication) [62].

The Bovine TB Stakeholder Forum [3] is an important national initiative, seeking broad stakeholder engagement in the future of the national TB eradication programme. Discussions are informed by the National Farmed Animal Health Strategy [63] which is underpinned by four key enabling principles (working in partnership, acknowledging roles and responsibilities, reflecting costs and benefits, applying the principle of ‘Prevention is better than cure’). Established in 2009, Animal Health Ireland (http://animalhealthireland.ie) provides one model where industry engagement has been facilitated in an Irish context.

## Conclusion

Based on current knowledge, it will not be possible to eradicate TB by 2030 with current control strategies plus badger vaccination. Additional measures will be needed if Ireland is to eradicate TB within a reasonable time frame. Adequate information is available, both from research and international experience, to indicate that these additional measures should broadly focus on adequately addressing TB risks from wildlife, implementing additional risk-based cattle controls, and enhancing industry engagement. Decisions made now will have long-term implications both in terms of time-to-eradication and cumulative programme cost.

## Footnotes

1 *Veterinary Microbiology*, Volume 40, issues 1–2, pp. 1–207 (1994)

2 *Tuberculosis*, Volume 81, issues 102, pp. 1–187 (2001)

3 *Veterinary Microbiology*, Volume 112, issues 2–4, pp. 89–394 (2006)

4 *Veterinary Microbiology*, Volume 151, issues 1–2, pp. 1–208 (2011)
